# 

**Published:** 1915-01-30

**Authors:** 


### CONTENTS.

| | PAGE |
|---|---|
| **Civil Hospitals and Military Officials** ... ... | 389 |
| **Pharmacopœial Doses** .. ... ... ... | 390 |
| **Hospital and Institutional News** ... ... ... | 391 |
| New Secretary of Hampstead General Hospital—Side Issues of the Appointment—Poor-Law Medical Officers and Clinical Work—The British Hospitals Association and Payment for Soldiers — Government Payment for the Wounded : An Edinburgh View—Staff Salaries and the War—American Women's War Hospital—A Chairman's Powers and a Secretary's Rights—From Laundry to X-Ray Department —The Chadwick Gold Medallists — The Chadwick Military Medallist—A Poor-Law Inspector on the Effect of the War—An Official Experiment with Clinical Assistants—The Japanese Red Cross Contingent : Arrival in London—Royal Visit to the Empire Hospital—The Burnley Hospital Competition—Hertford County Hospital Takes Our Advice—The Extent of War Hospital Accommodation—Hospitals for Postmen—The Condition of Convalescent Soldiers—A Doctor Sues a Colleague for Fees—The War and Hospital Architecture —The Insurance Act in London—Improvements at Colney Hatch Mental Hospital—This Week's Drug Market. | |
| **War and Disease** ... ... ... ... ... | 397 |
| The Russo-Japanese and South African Campaigns. | |
| **Welsh Hospital, Netley** ... ... ... ... | 398 |
| **I.—A Visit to Stockholm Hospitals** ... ... | 399 |
| By a Travelling R.M.O. | |
| **Round the World** ... ... ... ... ... ... | 401 |
| Fellow-Workers and the Roll of Honour. | |
| **Some Home-Made Hospital Equipment** ... | 402 |
| Examples from Leicester Infirmary : Heating Apparatus for Theatre—A Radiant Heat Novelty—Hospital Doors. | |
| **Clinical Records in Poor-Law Infirmaries** ... | 403 |
| A Rejoinder : By an Infirmary Medical Superintendent. | |
| **II.—Some Provincial Hospital Chapels** ... ... | 404 |
| Queen's Hospital, Birmingham. | |
| **The Editor's Letter-Box** ... ... ... ... ... | 405 |
| Damage to Hospitals by Hostile Air-craft. Section 12 of the National Insurance Act : A Year's Experience. | |
| **Workers' Acknowledgment to Sir Edward Wood** ... ... ... ... ... ... ... ... | 405 |
| **The Department of Modern Nursing** ... ... | 406 |
| VIII.—London Homœopathic Hospital. | |
| **London Panel Committee** ... ... ... ... | 408 |
| Discrepancies as to the Number of the Insured. | |
| **Glamorgan and Monmouth Hospital in France** | 408 |
| **The Institutional Worker** ... ... (Suppl.) | 1 |
| The Ventilation of Operating Theatres. Approved Homes. | |

